# Projected Evolution of California's San Francisco Bay-Delta-River System in a Century of Climate Change

**DOI:** 10.1371/journal.pone.0024465

**Published:** 2011-09-21

**Authors:** James E. Cloern, Noah Knowles, Larry R. Brown, Daniel Cayan, Michael D. Dettinger, Tara L. Morgan, David H. Schoellhamer, Mark T. Stacey, Mick van der Wegen, R. Wayne Wagner, Alan D. Jassby

**Affiliations:** 1 U.S. Geological Survey, Menlo Park, California, United States of America; 2 U.S. Geological Survey, Sacramento, California, United States of America; 3 Climate Research Division, The Scripps Institution of Oceanography, U.S. Geological Survey, La Jolla, California, United States of America; 4 Department of Civil and Environmental Engineering, University of California, Berkeley, California, United States of America; 5 UNESCO-IHE (Institute for Water Education, United Nations Educational, Scientific and Cultural Organization), Delft, the Netherlands; 6 Department of Environmental Science and Policy, University of California Davis, Davis, California, United States of America; Mt. Alison University, Canada

## Abstract

**Background:**

Accumulating evidence shows that the planet is warming as a response to human emissions of greenhouse gases. Strategies of adaptation to climate change will require quantitative projections of how altered regional patterns of temperature, precipitation and sea level could cascade to provoke local impacts such as modified water supplies, increasing risks of coastal flooding, and growing challenges to sustainability of native species.

**Methodology/Principal Findings:**

We linked a series of models to investigate responses of California's San Francisco Estuary-Watershed (SFEW) system to two contrasting scenarios of climate change. Model outputs for scenarios of fast and moderate warming are presented as 2010–2099 projections of nine indicators of changing climate, hydrology and habitat quality. Trends of these indicators measure rates of: increasing air and water temperatures, salinity and sea level; decreasing precipitation, runoff, snowmelt contribution to runoff, and suspended sediment concentrations; and increasing frequency of extreme environmental conditions such as water temperatures and sea level beyond the ranges of historical observations.

**Conclusions/Significance:**

Most of these environmental indicators change substantially over the 21^st^ century, and many would present challenges to natural and managed systems. Adaptations to these changes will require flexible planning to cope with growing risks to humans and the challenges of meeting demands for fresh water and sustaining native biota. Programs of ecosystem rehabilitation and biodiversity conservation in coastal landscapes will be most likely to meet their objectives if they are designed from considerations that include: (1) an integrated perspective that river-estuary systems are influenced by effects of climate change operating on both watersheds and oceans; (2) varying sensitivity among environmental indicators to the uncertainty of future climates; (3) inevitability of biological community changes as responses to cumulative effects of climate change and other drivers of habitat transformations; and (4) anticipation and adaptation to the growing probability of ecosystem regime shifts.

## Introduction

Planet Earth is warming at an accelerating rate. The latest assessments show the 2000s to be the third consecutive decade of record high global-average surface temperature [1], and 2010 tied with 2005 as the warmest year since records began in 1880 (http://www.ncdc.noaa.gov/sotc/global/2010/13). This warming is attributed with high probability to increasing human emissions of greenhouse gases [2]. Global warming has altered water supplies through changes in precipitation, evapotranspiration, runoff and river discharge [3]. Risks to coastal communities and infrastructure are growing as the rate of sea level rise accelerates [4] and as the intensity of tropical storms is projected to increase [5]. Surface temperatures of inland water bodies [6], rivers [7] and oceans [1] have all increased significantly. Warming of streams and rivers contributes to local species extinctions and facilitates colonization by introduced species [7]. Spring warming of temperate lakes disrupts the synchrony between zooplankton and their phytoplankton food supply [8]. Warming of the world oceans strengthens thermal stratification and has contributed to a 1% per year loss of oceanic primary production over the past century [9]. Therefore, evidence is accumulating on a global scale of strong links between climate warming and changes in availability of fresh water, risks to humans from coastal flooding and storms, and altered biological diversity and productivity of aquatic ecosystems.

Simulations with global climate models (GCMs) under a plausible range of greenhouse gas emissions scenarios all project substantial warming through the 21^st^ century [2]. Continued warming will have important consequences for social and natural systems, but these consequences will not be felt uniformly across the planet [1], [3], [6]. Therefore, strategies for adaptation to climate change require quantitative projections of how altered global patterns of temperature, precipitation and sea level will cascade to regional and local scales. We illustrate here one approach for developing quantitative projections by linking models of processes computed at sequentially smaller scales, from global to regional to local.

**Figure 1:**
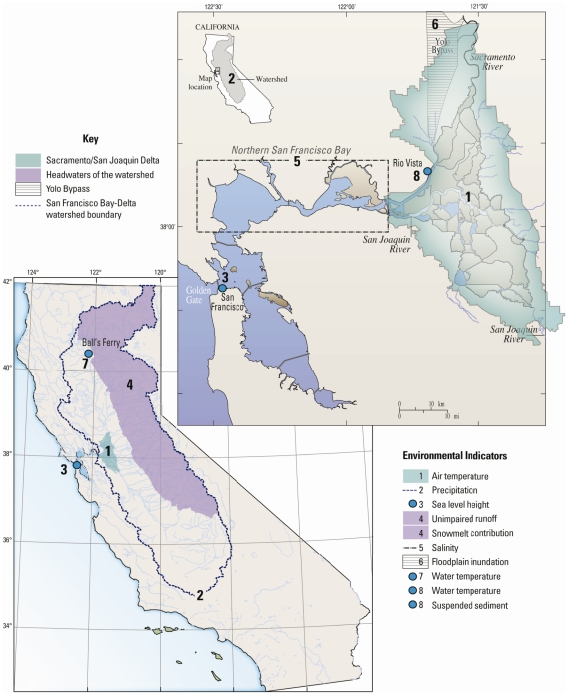
Spatial domains of environmental indicators. Shaded or hatched areas represent spatial domains of indicators representing areal averages or pertaining to a broad area, and blue dots represent locations of indicators corresponding to specific sites. Key shows geographic descriptions, and legend on lower-right shows corresponding indicators; compare to Figs. 2–3 and Table 1.

Our study is focused on California's San Francisco Estuary-Watershed (SFEW), which includes San Francisco Bay, the Sacramento-San Joaquin Delta (Delta) and the Sacramento and San Joaquin river drainages (Fig. 1). The SFEW has social and economic significance as the source of runoff that provides drinking water to 25 million people [10] and irrigation water to a million hectares of farmland producing crops valued at $36 billion per year [11]. It also has large ecological significance because the river system is habitat for native fishes including Pacific salmon and steelhead trout. San Francisco Bay is the largest estuary on the US west coast, providing habitat for endemic species (e.g. delta smelt, salt marsh harvest mouse) and marine species supporting fisheries (e.g. English sole, Dungeness crab). Fourteen species of migratory or Delta-resident fishes are imperiled, and their population declines motivate ambitious and costly programs of environmental conservation [12] and habitat rehabilitation [13]. On the shores of this estuary, 270,000 people and $62 billion of development are at risk of flooding as sea level continues to rise [14]. Regional planning and conflicts of resource allocation in the SFEW are already great challenges. These challenges are likely to grow as the regional effects of global climate change and other changes accumulate through this century. Here we develop integrated scenarios of the future SFEW by projecting a suite of environmental responses to climate change and assessing their implications for sustainability of native biota, water supplies, and risks of coastal flooding.

### Regional setting

The San Francisco Estuary-Watershed is composed of an interconnected airshed, watershed, river network, estuary and coastal ocean (Fig. 1). The 163,000-km^2^ watershed is bounded by the Sierra Nevada and Cascade mountains. Regional climate is characterized by a winter wet season and summer-autumn dry season. An average of forty percent of annual runoff to the river network is produced from snowmelt [15]. Reservoirs are managed to capture this late-season runoff as a resource, while water reaching the reservoirs during the earlier rainy season is managed as a hazard and allowed to pass through the reservoirs to maintain flood control space. Runoff and reservoir outflows collect in the Sacramento and San Joaquin Rivers, which converge in the Delta (Fig. 1). Tides propagate through the Golden Gate to the Delta, and the extent of salinity intrusion into northern San Francisco Bay is determined primarily by sea level height and river inflow. California's hydrology has followed the climate-driven patterns of change observed across the western United States and attributed to human-induced warming [16]. These patterns include trends of increasing winter and spring air temperatures and lengthened growing seasons [17], decreasing contributions of snow to annual precipitation [18], and advancement of spring snowmelt by 5 to 30 days [19]. Mean sea level at the entrance to San Francisco Bay has increased about 2.2 cm decade^−1^ since the 1930s, and the frequency of extreme tides has increased 20-fold since 1915 [20].

Future climates have been evaluated for the California region, where air temperatures are projected to increase 1.5 to 4.5°C this century in a range of scenarios [21]. Projected responses to warming include further declines of snow accumulation, decreasing hydropower generation, reduced viability of many species of fruit trees, high susceptibility of alpine and subalpine forests to warming, and increasing fire frequency [22]. Global sea level rise, expected to be a close index for that in California [20], is projected to be 70–185 cm above the present-day level [23]. Climate-driven changes in the California region are therefore expected to increase risks to the sustainability of native plant and animal communities and to human health, infrastructure, water supply and food production [24]. Here, we build from these past regional assessments to investigate how the combined effects of rising sea level and hydroclimatic changes could transform California's large watershed-river-estuary-ocean system through the 21^st^ century. Our projections suggest that climate-driven changes to the SFEW could require adaptations to an interconnected suite of responses including: a diminishing water supply, continued shifts toward wetter winters and drier summers, sea level rising to higher levels than were projected only a few years ago, salt water intrusion, reduced habitat quality for native aquatic species, and expanding envelopes of environmental variability into regimes we have not experienced. Adaptations to these responses would require integrated and flexible planning to cope with growing risks to humans and the increasingly difficult challenge of meeting demands for fresh water and sustaining native biota and their supporting ecosystem functions.

## Methods

We chose to evaluate two very different scenarios selected from the GCM projections used in the IPCC Fourth Assessment Report [2]. The PCM-B1 climate scenario portrays the B1 emissions scenario (representing a future where GHG emissions are curtailed by mid-century) as modeled by the Parallel Climate Model (PCM), a model with relatively low sensitivity to GHG emissions [25]. The GFDL-A2 climate scenario represents the A2 emissions scenario (corresponding to a future of continually increasing atmospheric greenhouse gases) as modeled by the medium-sensitivity NOAA Geophysical Fluid Dynamics Laboratory (GFDL) CM2.1 model [26]. These model-emissions scenario combinations were chosen to span a wide range of possible futures with regard to amount of warming and precipitation change, providing a comparison between a projection of a warmer future with little change in precipitation (PCM-B1) and that of a much warmer and drier future (GFDL-A2).

Our approach was to use linked models, each representing a different component of the system, to propagate the effects of the climate scenarios described above through the watershed-river-estuary system. Ultimately we portrayed these effects with a series of environmental indicators representing multiple components. These indicators were developed for the current century (2010–2099) and for a baseline period, defined as 1970–1999 to capture recent historical behavior (1999 is the end year of the “historical” GCM runs—see below). For all indicators, observation-based and model-based indicators were produced for the historical period to allow for model evaluation and to provide a baseline for assessing scenario projections.

**Figure 2:**
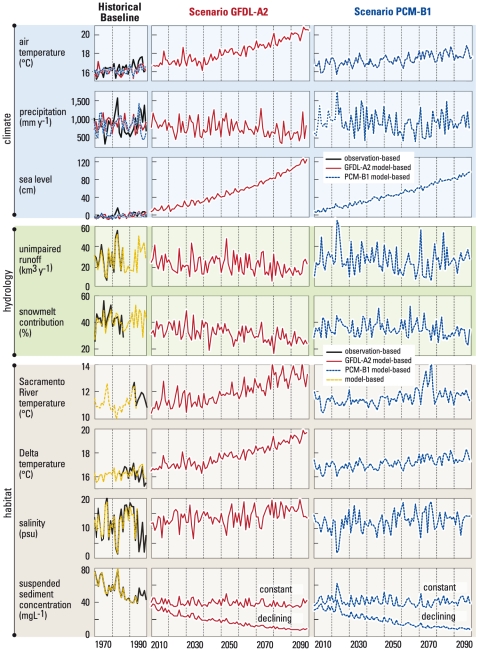
Projected 2010–2099 changes in annual mean values of nine environmental indicators for the A2 (red lines) and B1 (blue lines) scenarios compared to modeled and observed values during the 1970–1999 baseline period (left panels). The indicators measure changes in regional climate, regional hydrology, and habitat quality in the San Francisco Estuary-Watershed system. The GFDL-A2 and PCM-B1 “historical” data represent simulated realizations of possible climates constrained only by historical GHG forcing, and thus are not expected to track observed historical variability on a year-to-year basis.

For those indicators calculated directly from GCM output (air temperature, precipitation, and sea level), “historical” GCM simulations (driven by historical GHG forcings but otherwise unconstrained by observations) from the PCM and GFDL models were used to produce “model-based” historical indicators. Since the GCMs are freely running atmosphere-ocean-land models constrained only by observed GHG concentrations, these indicators will not agree on a year-to-year basis with the corresponding observation-based indicators (Fig. 2). Thus, the GCMs should be evaluated based on their statistical agreement with the observations, including model bias and variance. The model historical measures are essential to provide a baseline against which to compare the corresponding projections.

For indicators derived from the chain of models downstream from the GCMs, the model-based historical indicators are ultimately based on observed meteorological forcings, but they also reflect errors introduced by the linked models used to produce them. As such, these indicators allow for direct model evaluation by comparison with the corresponding “observation-based” time series, as well as providing a model-based baseline against which to compare the projections.

**Figure 3:**
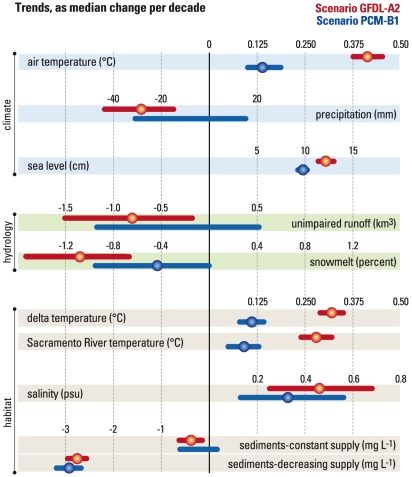
Projected 2010–2099 changes in nine environmental indicators, expressed as median trend per decade, for the A2 scenario (red) and B1 scenario (blue). Statistically significant (p<0.05) trends are indicated with solid circles; horizontal lines show 95% confidence limits of the trend estimates.

The trend slope for each indicator time series (Fig. 3) was calculated using the approach of Theil [27] and Sen [28]. Trend significance was determined using the modified Mann-Kendall approach of Yue and Pilon [29] which corrects for serial correlation. The confidence interval on the trend was calculated using the method described by Sen [28].

Descriptions of the individual component methods follow. An expanded methods section is in Supporting Information (Methods S1).

### Meteorology

Daily values of the climate variables for the GFDL-A2 and PCM-B1 climate scenarios, and for historical PCM and GFDL model runs (forced using historical GHG concentrations) were obtained from the Program for Climate Diagnosis and Intercomparison at the Lawrence Livermore National Laboratory ([30]; www-pcmdi.llnl.gov). The GCM simulations were made on global grids with about 2 to 3° latitude and longitude resolution (about 250 km at the latitude of the Delta), and thus the original GCM scenarios were too spatially coarse for the purposes of this study. The GCM temperatures and precipitation values were downscaled onto a 1/8° latitude-longitude grid over the study area by a method called Constructed Analogs [31]. This method is designed to ensure that daily weather simulated by the GCM is consistently carried down to the 12-km scale, and also to yield realistic temperatures across areas with sharp geographic gradients, as in California. The method was applied to climate simulations spanning the period from 1970–2099 to obtain daily, gridded temperature and precipitation patterns over California, from which watershed- and Delta-average (see Fig. 1) values were extracted. The corresponding averages based on historical observations were derived from the gridded meteorological dataset of Maurer et al. [32].

### Sea level

A model [20] was adopted to investigate sea level trends and extremes. The model was trained from historical data and used to project future water levels at the San Francisco Golden Gate tide gage location (Fig. 1). The model consists of four components: predicted astronomical tides, synoptic meteorologically-forced sea level fluctuations (based on local sea-level pressure and regional wind stress), ENSO-related monthly-to-interannual fluctuations, and long-term sea level rise associated with global warming. The synoptic and ENSO components were produced with regression models based on historical data [20] and applied to GCM outputs. The climate-change component was based on the method of Vermeer and Rahmstorf [23]. Simulated sea level at the Golden Gate was constructed by superposing these four components, yielding a time series of hourly sea levels from 1970 through 2099 for each climate scenario. Historical observations for 1970–1999 were obtained for the Golden Gate tide gage from NOAA (tidesandcurrents.noaa.gov).

### Hydrology and management

A combination of models was used to simulate the watershed's hydrologic behavior. Downscaled meteorological fields (see “Meteorology” above) were used to drive the VIC watershed model [33], [34], configured for the Sacramento River and San Joaquin River watersheds using the same parameters applied in several prior studies of the area [16], [21], [35]. This resulted in daily estimates of unimpaired reservoir inflows for each scenario. A simulation was also performed for the baseline period, driven using historical meteorology [32] to produce the model-based historical hydrological indicators. Estimates of unimpaired flow at major reservoirs throughout the watershed were obtained from the California-Nevada River Forecast Center (www.cnrfc.noaa.gov) and the California Data Exchange Center (CDEC, cdec.water.ca.gov). Data covering the period 1970–1986 were available, allowing total watershed unimpaired runoff and snowmelt fraction of annual runoff to be calculated for this period, providing the observation-based historical time series for those indicators.

These inflows were used to drive a model of freshwater management operations—the California Department of Water Resources' CALSIM II model [36]. CALSIM is a management optimization model in which, given inputs of reservoir inflows, a set of freshwater management decisions is determined at each time step that optimally satisfy operational goals and constraints. The results are estimates of managed freshwater flows at points throughout the watershed. CALSIM has been applied in other climate-change studies [37], [38], [39], [40], [41]. In this study, a new configuration of CALSIM II was used to produce projections for the coming century, and an existing configuration (configured for runs only up to 1994) was used to produce historical estimates (1970–1994). Finally, monthly historical and projected stream temperatures were simulated throughout the watershed using the U.S. Bureau of Reclamation's CALSIM-driven stream-temperature model. This model has also been applied in other climate-change studies (e.g., [40]).

### Estuarine salinity

Two complementary models were used to project changes in estuarine salinity due to climate change. The Uncles-Peterson (U-P) model, a 2D box model of San Francisco Bay (Fig. 1), accurately reproduces salinities at weekly to interannual time scales over a wide range of flow regimes [42], [43]. Importantly, the U-P model is very economical computationally, enabling the 90-year runs needed to evaluate estuarine variability under the climate scenarios. The U-P model was driven using daily freshwater inflows derived from CALSIM outputs described above, producing daily salinities along the estuary's axis for the historical baseline period and for each future scenario. A simulation was also performed for the baseline period using observed inflows (www.water.ca.gov/dayflow) to derive “observation-based” historical salinity values.

While the U-P simulations provide a representation of the influence of changing upstream hydrology on estuarine salinities, the U-P model does not capture the effects of sea level rise on salinity. The Delft3D model of San Francisco Bay [44] is a 3D process-based model that is sophisticated enough to capture these effects. Delft3D is, however, too computationally demanding to evaluate full 90-year scenarios, and was thus applied in a complementary manner with the U-P model. Multiple runs of Delft3D were used to develop a regression model of salinity changes based on amount of sea level rise (see Supporting Information, Methods S1 for details), which was then driven by historical values of mean sea level for the baseline period, and by sea level projections through the end of this century (see “Sea level” above). The changes were added to the corresponding U-P salinities, and the final results represent our estimate of salinity changes throughout the estuary due to the combination of upstream hydrologic forcing and sea level rise.

### Suspended sediment

To evaluate suspended sediment changes under the climate-change scenarios, we developed a rating curve of suspended sediment concentration (SSC) at Rio Vista (Fig. 1) versus Sacramento River discharge (Fig. S1). For each scenario, daily discharges (see “Hydrology and management” above) were used to calculate the daily median SSC, which was then annually averaged. Sediment delivery from the Sacramento River watershed to San Francisco Bay has decreased by about one-half between 1957 and 2001 [45]. As these changes in sediment delivery have occurred, the turbidity and associated SSC within the Delta have also decreased by approximately 40% (Fig. S2). Because it is unclear whether this trend will continue, we developed two sediment-supply scenarios (Fig. S3). The first scenario assumes that the historical rating curve applies in the future, and the second assumes that SSC decreases at 1.6% yr^−1^, the Delta-wide average rate of SSC decrease from 1975–2008 (data from the Interagency Ecological Program's Environmental Monitoring Program at www.water.ca.gov/bdma; Seasonal Kendall test [46]). Since little observed SSC data exist for the baseline period, the rating curve was applied to produce a hindcast of SSC, using observed discharges (www.water.ca.gov/dayflow) and the historical trend in sediment delivery. This is presented in Fig. 2 as the “observation-based” time series of SSC during the baseline period. The historical “model-based” indicator was produced by applying the rating curve to the CALSIM-based daily discharge estimates (see “Hydrology and management” above), and using the historical trend in sediment delivery.

### Delta water temperature

Water temperature data were obtained from the Interagency Ecological Program for the Sacramento River at Rio Vista, where water temperatures were collected from May 1983 through September 2002 (1984–1999 annual averages of these data constitute the observation-based historical baseline). Historical air temperature and insolation data were also acquired (www.cimis.water.ca.gov, www.calclim.dri.edu/data.html). A regression was developed to relate the daily-averaged water temperature to the air temperature and insolation from the same day and water temperature from the preceding day [47]. To project water temperatures for the coming century, the model was applied to the downscaled climate data (see “Meteorology” above), using the mean annual insolation cycle. Similarly, to hindcast water temperatures for 1970–1999, the model was forced with the long-term historical air temperatures and the mean annual insolation cycle, providing the “model-based” historical indicator for Delta water temperature. Annual averages were calculated from the daily model output (see Methods S1 for additional discussion).

### Biological indicators

Delta smelt (*Hypomesus transpacificus*) is endemic to the San Francisco Estuary [48], [49]. It is listed as endangered by the state of California, and a change in status from threatened to endangered has been deemed warranted under the US Endangered Species Act. Thus, maintaining the population of delta smelt has become a key goal in managing the estuary [50]. To assess the effects of climate change on delta smelt, the frequency of mean daily water temperatures above 25°C was determined from modeled water temperatures at Rio Vista (see “Delta water temperature” above), a location within one tidal excursion of a large portion of delta smelt habitat in the Sacramento River. Multiple studies indicate that mean daily temperature of 25°C is a threshold for high mortality of delta smelt [48], [51], [52].

Winter-run Chinook salmon (*Oncorhynchus tshawytscha*) is endemic to the Sacramento River system of California and is listed as endangered under both state and US endangered species legislation [49]. Most of the population is subject to water temperature regulation by Shasta Reservoir. Winter-run Chinook salmon begin spawning in the spring. Developing embryos and pre-emergent fry are expected to be in the gravel from May through October. The effects of climate change on winter-run Chinook salmon were assessed by comparing projected mean monthly water temperatures (see “Hydrology and management” above) for the period May–October against a threshold of 16°C, which would result in high mortality of eggs and pre-emergent fry. This is likely a conservative comparison since in a month with a mean of 16°C, approximately half the days would have higher temperatures. Comparisons were made for the Sacramento River at Balls Ferry (Fig. 1), which is at the lower end of the spawning reach. Historical temperature data were obtained for 1991–1999 from CDEC and were used to produce the corresponding observation-based historical indicator. Stream temperature data from the historical run of the stream temperature model (1970–1994; see “Hydrology and management” above) were used to produce the model-based historical indicator.

Sacramento splittail (*Pogonichthys macrolepidotus*) is a large cyprinid, endemic to the San Francisco estuary and watershed [49], [53]. Splittail are true floodplain spawners and production of strong year-classes is associated with flooding of Sutter and Yolo bypasses, floodways designed to protect urban areas from flooding. Yolo Bypass (Fig. 1) provides benefits to native fishes, including Chinook salmon and splittail [54]. Floodplains must remain continuously flooded for a minimum of about 30 days [55] for splittail to successfully spawn, and longer inundation periods result in greater production of young splittail [53]. Yolo Bypass provides appropriate spawning conditions at flows above about 113 m^3^ s^−1^. Therefore, for each scenario we counted the number of floods each year in which flows continuously exceeded 113 m^3^ s^−1^ for at least 30 days.

## Results

### Projected responses to climate change in the 21^st^ Century

Our objective was to develop quantitative visions of the SFEW system in two contrasting future climates and to communicate those visions in a way that makes them useful for planning adaptation strategies. Therefore, from the many outputs of models described above we selected nine (Table 1) to use as indicators of changing climate, hydrology and habitat quality. The climate indicators are air temperature over the Delta, precipitation over the Sacramento-San Joaquin River basin, and water elevation at the entrance to San Francisco Bay (Fig. 1). Hydrologic indicators, modeled using the climate projections as inputs, are unimpaired runoff from the headwater basins of the Sierra Nevada and Cascade ranges and the snowmelt contribution to runoff. Habitat indicators, modeled using the climate and hydrologic projections as inputs, are salinity in northern San Francisco Bay, water temperature in the upper Sacramento River, and water temperature and suspended sediment concentration (SSC) in the Delta (Fig. 1). We show future visions of the SFEW as yearly mean values of each environmental indicator for the period 2010–2099 and compared to the 1970–1999 baseline period (Fig. 2). To simplify presentation of results we use “B1 scenario” to denote projections from the PCM model using B1 GHG emissions, and “A2 scenario” to denote projections from the GFDL model using A2 emissions.

**Table 1:** Environmental indicators analyzed directly (top 10; see Figs. 2–3) or exceedences of thresholds (bottom 4; see Fig. 4), with corresponding spatial domains (see Fig. 1), units of measurement, and social/ecological significance.

Indicator	Spatial Domain	Metric	Significance
Air temperature	Sacramento-San Joaquin Delta	°C (annual mean)	Water supply; water & habitat quality; human health
Precipitation	Sacramento-San Joaquin watershed	mm yr^−1^	Water supply; water & habitat quality
Sea level height	San Francisco Bay entrance	cm	Flood risk; water & habitat quality
Unimpaired runoff	Sacramento-San Joaquin headwaters	km^3^ yr^−1^	Water supply; flood protection; reservoir operations; water & habitat quality
Snowmelt contribution	Sacramento-San Joaquin headwaters	percent (of annual runoff)	Seasonal hydrology; flood protection; water & habitat quality
Salinity	Northern San Francisco Bay	psu (April–June mean)	Estuarine habitat quality; drinking-water quality
Water temperature	Upper Sacramento River	°C (annual mean)	Habitat quality
Water temperature	Sacramento-San Joaquin Delta	°C (annual mean)	Habitat quality
Suspended sediment - constant supply	Delta, Lower Sacramento River	mg L^−1^ (annual mean)	Habitat & water quality; estuary geomorphology; wetland sustainability
Suspended sediment - decreasing supply	Delta, Lower Sacramento River	mg L^−1^ (annual mean)	Habitat & water quality; estuary geomorphology; wetland sustainability
Extreme water level	San Francisco Bay entrance	h yr^−1^>99.99th percentile	Flood risk
Lethal water temperature	Upper Sacramento River	months yr^−1^>16°C	Sustainability of winter-run Chinook salmon
Lethal water temperature	Sacramento-San Joaquin Delta	days yr^−1^>25°C	Sustainability of delta smelt
Floodplain inundation	Yolo Bypass	flow>113 m^3^ s^−1^, duration>29 d	Ecosystem restoration (floodplain habitat management)

Most indicators show good agreement between historical model-based and observation-based time series (Fig. 2, left panels). The climate indicators are not necessarily expected to agree in this sense because the “historical” GCM runs do not correspond to actual historical variations, but instead reflect a realization of climate given historical GHG forcings. Of the remaining indicators, Sacramento River water temperature has only three years of overlap between observations and simulations, though agreement is good during that time. Annually averaged Delta water temperature shows poor agreement (r = 0.41) during the period shown. This is a result of three high-flow years near the end of the comparison period, which cause errors in the annual averages (see Methods S1 for more details). The effect of high flow on Delta temperatures (Fig. S4) does not create significant biases in the projections because unimpaired runoff changes little (B1) or declines (A2) for the climate scenarios presented. At the daily timescale, which is critical to fish survival, the comparison of modeled and observed temperatures yielded very high correlations (r = 0.98). Unimpaired runoff, snowmelt fraction of annual runoff, north Bay salinity, and suspended sediment concentrations all have high correlations (r = 0.99, 0.87, 0.98, and 0.997, respectively) that are strongly statistically significant (p<0.00001).

Air temperature increases steadily in both future scenarios (Fig. 2), but the rate of change is faster in the A2 scenario (maximum annual temperature reaches 21°C) than in the B1 scenario (maximum annual temperature of 18.6°C). Annual precipitation declines steadily in the A2 scenario and is persistently below the modeled 1970–99 baseline by the latter part of the century. There is no apparent secular trend of precipitation change in the B2 scenario, but this projection has large interannual variability that includes years of extreme high precipitation and a simulated multi-year drought in the 2070 decade (Fig. 2). These two future climates span much of the range of temperature and precipitation projections made within a larger ensemble of climate models and GHG emissions [21]. Our projections of sea level rise are within the range of global sea level rise developed in recent studies [4] and reach 125 cm (A2) and 96 cm (B1) above the observed and modeled baselines by the end of this century (Fig. 2).

The hydrologic indicators reflect combined effects of changing air temperature and precipitation. Projections of unimpaired runoff largely reflect changes in precipitation. Runoff in the A2 scenario is 11–12% below the baseline during the first two–thirds of the century. Then, coincident with the simulated end-of-century drought, runoff drops another 16% and persists at this low level for nearly 15 years. Runoff in the B1 scenario exhibits the same large interannual variability of precipitation, including an extremely wet year in 2023 and two very wet years and large droughts between 2065 and 2085. The snowmelt contribution to annual runoff declines steadily in the A2 scenario, but it shows no obvious trend in the B1 scenario until the last two decades when runoff is consistently below the historical mean (Fig. 2). These changes imply continuing shifts toward earlier runoff as a declining fraction of annual runoff occurs during the snowmelt season.

We used these climate and hydrologic projections to develop the first quantitative assessments of how habitat quality in the SFEW will be altered by climate change. As a response to both sea level rise and reduced runoff, computed salinity in northern San Francisco Bay increases 4.5 (A2 scenario) and 2.2 psu (B1 scenario) above the 1979–1999 baseline during the last third of the century. Mean annual water temperature in the upper Sacramento River approaches or exceeds 14°C regularly toward the end of the A2 scenario, and also during the projected 2070s drought in the B1 scenario. Delta water temperatures also increase steadily in both future climates, most rapidly in the A2 scenario. Suspended sediment concentrations in the Delta were calculated as a function of river inflow, assuming that either (a) the supply of erodible sediments in the river system remains constant, or (b) supply decreases as the declining trend of recent decades [56] continues. Sediment concentrations decline slightly under assumption (a), but rapidly under assumption (b) in both climate scenarios (Fig. 2).

We emphasize that such model-based projections are not predictions but instead are plausible depictions of how this complex landscape might respond to prescribed model- and emissions-specific future climates. Importantly, we have not considered potentially confounding effects of changing water resource management objectives, rules or infrastructure. We also have not considered changes in land use or infrastructure that might occur through planned actions or catastrophic events such as major levee breaks. However, even considering these constraints and caveats, our projections from two different climate scenarios include years with mean air and water temperature, sea level height and estuarine salinity well above observed and modeled values in the 1970–99 baseline period (Fig. 2). They also include years with annual precipitation, snowmelt contribution to runoff and suspended sediment concentrations well below modeled and observed historical values.

### Trends of the environmental indicators

Indicators of climate-driven environmental change will be most useful to policy makers and resource managers if they measure rates of change and indicator sensitivity to different climate scenarios. We extracted this information from the time series of each indicator shown in Fig. 2 by calculating an overall trend for the period 2010–2099 and measuring its statistical significance. The trends represent median rates of change over the 90-year series, and are expressed as rates of change per decade. Results in Fig. 3 present an integrated view of how the SFEW system will respond to global climate change as realized in two future scenarios. Among the climate indicators, air temperature and sea level increase significantly in both scenarios. Air temperature increases 0.42°C decade ^−1^ in the A2 scenario, but only 0.14°C decade^−1^ in the B1 scenario (Fig. 3). Sea level increases 12.3 and 9.9 cm decade^−1^ in the A2 and B1 scenarios, respectively. Precipitation declines significantly (−28 mm decade^−1^) in the A2 scenario, but does not have a significant trend in the B1 scenario. The hydrologic indicators respond to these changes in precipitation and air temperature. Unimpaired runoff, like precipitation, has a significant negative trend in the A2 scenario (−0.80 km^3^ decade^−1^) but not in the B1 scenario (Fig. 3). However, the snowmelt contribution to runoff declines significantly in both scenarios, at −1.1% decade^−1^ (A2 scenario) and −0.4% decade^−1^ (B1 scenario).

Water temperatures in the Sacramento River respond to two factors, both of which trend significantly: 1) increasing air temperature, and 2) decreasing snowmelt runoff reducing the amount of cold water in the upstream reservoirs available to manage downstream temperatures. Water temperatures in the Delta, well removed from the effects of the major reservoirs, respond primarily to increasing air temperature. Sacramento and Delta water temperatures increase significantly, and at roughly the same rate, in both scenarios (Fig. 3). Salinity in northern San Francisco Bay (Fig. 3) also increases significantly in both scenarios (+0.46 psu decade^−1^ for A2, +0.33 psu decade^−1^ for B1), due to sea level rise in both scenarios and the added effect of declining runoff in A2. Suspended sediment concentrations in the Delta change only slightly if sediment supply in the river system remains constant, but they fall rapidly (−2.7 and −2.9 mg L^−1^ decade ^−1^) in both climates if sediment supply continues to decline. Therefore, projections of suspended sediment concentrations in the Delta, and consequently sediment transport to San Francisco Bay, are driven more by prescribed changes in sediment supply than by climate-driven changes in river discharge (Fig. 3).

### Increasing frequency of extreme events

**Figure 4:**
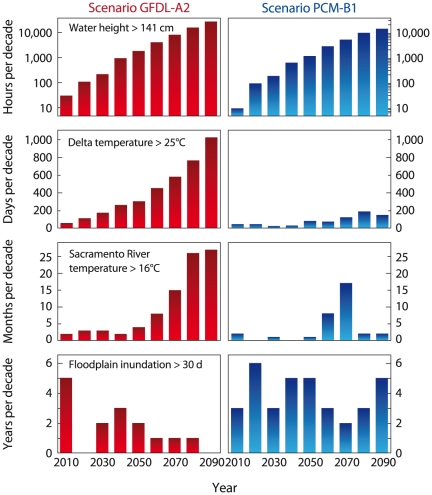
Projected 2010–2099 changes in the occurrence of extreme environmental conditions in the San Francisco Estuary-Watershed system for the A2 (left) and B1 (right) scenarios. The indicators count projected exceedences each decade of threshold values based on historical extreme water elevation or having significance for sustainability of native species of fish (lethal water temperatures) or habitat restoration through management of floodplain habitats.

Some important ramifications of climate change are not captured in annual mean indices because these don't depict changes in the frequency of extreme events [3]. We computed four environmental indicators as exceedence frequencies of threshold values chosen to measure risks to humans or native biota. Projected water levels at the Golden Gate were compared to the historical 99.99^th^ percentile of water elevation (141 cm, relative to the recent historical mean sea level). Both climate scenarios project marked increases in the frequency of extreme water heights over the historical rate of approximately 8 hours decade^−1^, amounting to increases to 2,000 (A2) and 1,200 (B1) hours decade^−1^ by mid century, and 30,000 (A2) and 15,000 (B1) hours decade^−1^ by the end of the century (Fig. 4).

As an indicator of habitat quality for delta smelt, we calculated number of days each decade when projected water temperature in the Delta exceeds 25°C. The frequency of occurrence of temperatures greater than 25°C increases gradually in the B1 scenario but rapidly in the A2 scenario (Fig. 4). The frequency of occurrence of lethal temperatures for Chinook salmon (>16°C) grows modestly in the B1 scenario, except during the simulated drought of the 2070-decade when this threshold is exceeded in 17 months (Fig. 4). River temperatures above 16°C become common (>20 months decade^−1^) after 2080 in the A2 scenario. The final habitat indicator is number of years each decade in which spring floods are large enough to inundate the Yolo Bypass (Fig. 1) for at least 30 consecutive days, a minimum threshold for successful spawning of Sacramento splittail. Spring flooding continues through the 21^st^ century in the B1 scenario. But the warmer and drier climate in the A2 scenario reduces the frequency of spring floods having duration long enough for successful spawning and rearing of this species (Fig. 4).

## Discussion

California's San Francisco Estuary-Watershed system is the focus of continuing policy debates centered around the challenge of meeting multiple and sometimes conflicting objectives of resource management [57] . Our projections show how those conflicts and the challenge of resource management could intensify as the water supply, sea level, and habitats are transformed by global climate change. We highlight five conclusions that emerge from our study, and end with general lessons to guide strategies of climate-change adaptation in this and other coastal landscapes.

### Uncertainty about how SFEW will evolve in the future

The two scenarios used in this study were chosen to explore possible futures and, at the same time, illustrate uncertainty. Different projected futures arise from differences among GCMs in their sensitivity to greenhouse gas emissions and from a range of possible GHG emissions trajectories. Propagation of this uncertainty into the physical and biological systems in SFEW varies among environmental indicators that fall into two classes. First are those with non-significant trends in the B1 scenario, but with large and significant trends in the A2 scenario: precipitation and unimpaired runoff (Fig. 3). Future changes of these indicators will depend on how much climate change is realized and thus on how sensitive the climate system proves to be to greenhouse gases and how future emissions evolve—neither of which can be predicted yet. If realized, the significant trends toward reduced precipitation and runoff in the A2 scenario would have important implications for California's future water supply. The second class of indicators includes those with significant trends in both scenarios, indicating that these represent likely regional responses to global warming. Within this class are two subclasses having different sensitivities to the uncertainty of climate projections. The projected trends of salinity increase, snowmelt decline, and SSC with decreasing supply have comparable magnitudes (overlapping confidence intervals) in the A2 and B1 scenarios (Fig. 3). Therefore, changes in these indicators are relatively insensitive to the uncertainty arising from differences among GCMs and emissions trajectories. The other subclass includes trends of air and water temperature and sea level, which have non-overlapping confidence intervals in the two scenarios. Therefore, changes in these indicators are likely, but the rates of change are strongly tied to projected rates of global warming, so these indicators are particularly sensitive to model- and emissions-specific scenarios.

This classification of projected responses to climate change suggests that regional planners and resource managers should consider: (a) strategies for adaptation to progressively increasing air and water temperature, sea level and salinity intrusion in the SFEW, and further shifts toward more runoff in winter and less in spring-summer; but (b) planning for a broad range of future water supply because GCMs differ widely in their projections of precipitation trends. Effective strategies will be flexible and responsive to new data and assessments of climate change as they emerge [58]. For example, projections of global sea level rise are evolving rapidly [4], [23] and are likely to undergo further revisions in the future. Therefore, our projections of environmental change are best viewed as a starting place; each will be modified as new information and tools emerge for assessing regional responses to global change [3].

### Today's extremes could become tomorrow's norms

These projections highlight an important manifestation of climate change: changes in mean values of hydroclimatic variables can induce relatively large changes in the frequency of extreme events [3]. As examples, we show projections of increasing frequency of exceptional sea level and water temperature in both scenarios, and of decreasing floodplain inundation in the A2 scenario (Fig. 4). These imply growing risks of coastal flooding, extinction of native fishes, and decreasing feasibility of some ecosystem restoration actions. Therefore, regional resource planning and risk assessments should anticipate shifts into regimes of environmental conditions unprecedented in the period of our social and economic development. This challenge is daunting because of large uncertainty reflected in the variability among indicators in their sensitivity to climate scenario (Fig. 4), and because changing frequency of extreme conditions implies that the indicators will fluctuate within new envelopes of variability over time – i.e., their underlying drivers become non-stationary. Today's resource-management tools are grounded in the assumption of stationary processes of natural variability. Climate change undermines that assumption [3], so adaptation will require development of new probabilistic models to assess environmental changes and their uncertainty in a nonstationary world.

### It's not just climate change

Our projections illustrate how responses to climate change could transform the SFEW into a very different system by mid-century (Fig. 2). Transformative change is not new to this ecosystem, which has been altered over the past 150 years by massive landscape modifications, water development, pollutant inputs and introductions of alien species [59]. We selected SSC as one example of an environmental indicator that is more sensitive to landscape change than to climate change. Cessation of hydraulic mining, flood management, and damming the large rivers have decreased sediment delivery to the estuary by about half [56]. Whether this decline continues or abates will have a much greater effect on the future trajectory of SSC than climate change (Fig. 2). This trajectory has important ecological implications because further reductions in sediment supply will increase vulnerability of tidal marshes and mudflats to sea level rise [60], reduce habitat quality for some native fishes, and might promote blooms of toxic cyanobacteria [61] that will be increasingly favored as nutrient-enriched Delta waters warm [62]. Assessments of climate-change impacts must therefore be placed in the broad context of all the drivers that will continue to transform coastal ecosystems [60], including population growth and urbanization, nutrient enrichment, catastrophic levee failures from storms or earthquakes, modified reservoir operations and water conveyances, and implementation of ecosystem restoration plans. Planning will be most challenging with regard to environmental indicators, such as sediment supply, which contain uncertainties in their responses to both climate change and these other drivers of change.

### Biological community changes are inevitable

Programs of biodiversity conservation will face an increasingly difficult challenge as environmental conditions in the SFEW diverge from those to which its native species are adapted [13]. Expected outcomes include increasing extinction risk of native species and continuing emergence of nonnative species as dominant components of biological communities. Fishes endemic to the Delta, such as delta smelt, are adapted to cool, turbid, low-salinity habitats [63]. Sustaining populations of these species will become increasingly difficult as Delta waters warm, clear and become more saline (Fig. 2). Of the four runs of Chinook salmon that spawn in the Sacramento-San Joaquin drainage, the winter run is at exceptional risk because its spawning is timed such that eggs develop in summer, when projected river temperatures reach lethal levels (Fig. 4). Communities of fish and their zooplankton prey in the Delta have become increasingly dominated by nonnative species whose successful invasions have been facilitated by synergistic effects of climate anomalies (extended drought) and flow management [64]. Our projections include significant departures from the contemporary climate and flow regimes in the future, so environmental conditions might continue to move toward those that select for nonnative biota.

We have learned from other studies that small perturbations can trigger ecosystem regime shifts. A recent example occurred in Denmark's Ringkøbing Fjord, where mean salinity increased 1.6 psu after actions were taken to enhance water exchange with the North Sea. This small salinity change was followed by sudden and unanticipated reorganization of biological communities at all trophic levels, from phytoplankton to macrobenthos and waterbirds [65]. We project larger salinity increases in San Francisco Bay by the end of the 21^st^ century (Fig. 2). Therefore, conservation plans should expect surprises and include monitoring to detect and contingencies for adapting to unexpected shifts in habitats and their biological communities. And, they should be designed to accommodate a range of future climates. Feasibility and outcomes of proposed habitat restoration actions, such as creation of seasonal floodplain habitat (Fig. 4), low-salinity aquatic habitats and thermal refugia for native species [13], will be very different as seasonal hydrology and water temperature change.

### The challenge of meeting California's water demands will intensify

California's water supply (annual unimpaired runoff) is projected to decline or remain steady (Fig. 3), and demands are likely to increase as populations and temperatures rise. Deficits of surface runoff are now met with groundwater pumping. However, pumping between 1998 and 2010 depleted 48.5 km^3^ of water from the Central Valley groundwater system, and continued groundwater depletion at this rate is unsustainable [66]. Future strategies of water management will require adaptations such as aggressively increasing water-use efficiency, reducing surface water deliveries, capturing more runoff in surface storage or groundwater recharge, and implementing programs of integrated regional water management [67]. Model results suggest that the inherent large annual variability of precipitation will persist (Fig. 2), even as longer-term trends of warming and possibly drying take hold. Therefore, water-resource planning should also include contingencies for longer dry seasons, extended droughts, and extreme floods due to shifts from snow to rain. Diminishing snow packs result in earlier reservoir inflow, so reservoir operations must adapt to a shift toward more water being managed as a hazard (flood control) and less as a resource (reservoir storage). Additional freshwater releases to mitigate increased salinity intrusion into the estuary will be required to maintain quality of drinking water to communities that use the Delta as their municipal water supply. These adaptations to maintain water supply for human consumptive uses will potentially constrain availability of water to meet objectives of habitat conservation plans, such as restoring natural flow and salinity variability to promote recovery of native biota in the Delta [13].

### General lessons to guide climate-change adaptation planning

To our knowledge, this is the first attempt at an integrated quantitative assessment of how global signals of climate change would cascade to modify runoff, river discharge, water temperature, sea level, salinity intrusion and suspended sediments in a large watershed-river-estuary-ocean system. Although our projections of climate-driven change are specific to SFEW, lessons from this place-based study can be used as a starting place to guide adaptation strategies elsewhere:

1. Outputs from complex models can be explored by simplifying into a small set of environmental indicators chosen to develop an integrated view of how climate change will be manifested across landscapes.
2. Climatic, hydrologic and habitat indicators vary in their sensitivity to uncertainty about the future; measures of that sensitivity provide important information for assigning priorities and including contingencies in adaptation planning.
3. Results from climate simulations and resulting assessments of climate-change impacts will continue to evolve as the underlying science improves, so adaptation planning must be responsive to the continuing emergence of new models, analyses and insights.
4. Assessments of climate-change impacts are best placed in the broader context of all the drivers of change because some environmental indicators are more sensitive to other drivers such as landscape transformations, species introductions, pollution and water development.
5. Biological community changes are inevitable, and programs of ecosystem rehabilitation and biodiversity conservation will be most likely to meet their objectives if they are designed from projections of the future climate rather than today's climate.
6. Environmental planning should anticipate and adapt to ecosystem regime shifts; monitoring is essential for detecting and responding to regime shifts.
7. Warming in regions such as the western United States implies that sustainability of reliable water supplies will require changes in water management. These adaptations will potentially exacerbate conflicts of water allocation to meet human demands and goals of biological conservation plans.

Finally, our results are consistent with other model-based projections that California's climate will continue to warm through the 21^st^ century. There is uncertainty about how much global temperature will rise in response to increases in greenhouse gases, but it is clear that the rate of warming will increase with higher greenhouse gas emissions [21], [24]. Environmental indicators considered here respond more rapidly and more strongly to the A2 scenario than to the B1 scenario (Figs. 2, 3). Collectively, these indicators depict climate-driven changes in the reliability of California's water supply, in risks to humans and ecosystems due to coastal flooding, and in likely outcomes of ecosystem restoration programs. Contrasting futures in the A2 and B1 scenarios show that mitigation steps that slow greenhouse gas emissions in the first half of the 21^st^ century would reduce the requirements for adaption to climate-change impacts through the end of the century. However, regardless of the greenhouse gas emissions trajectory, substantial global and regional warming is likely, so successful climate-change adaptation will require other near-term mitigation actions aimed at buffering some of the long-term climate-change effects depicted by our indicators.

## Supporting Information

Figure S1 **Sediment rating curve for the Sacramento River at Rio Vista, 1998–2002.**
(TIF)

Figure S2 **Mean annual turbidity, declining throughout the Sacramento-San Joaquin Delta from 1975–2008.** From monthly data provided by California Department of Water Resources, Environmental Monitoring Program.
(TIF)

Figure S3 **GFDL and PCM scenarios for suspended sediment concentration (SSC) in the Sacramento River at Rio Vista for constant and decreasing sediment supply.** Each band represents the interquartile range of SSC.
(TIF)

Figure S4 **Effects of high river flows on errors in modeled annual average Delta water temperatures.** Difference between modeled and observed yearly average water temperature is compared to the annually averaged Sacramento River flow; model-observation deviations occur in years with high river flow.
(TIF)

Methods S1 **Expanded description of methods with supporting references.**
(RTF)
