# Supplementary figures and images for: Projected Evolution of California's San Francisco Bay-Delta-River System in a Century of Climate Change
Source: PLoS One. 2011 Sep 21;6(9):e24465. doi: 10.1371/journal.pone.0024465 (PMC3177826; doi:10.1371/journal.pone.0024465)

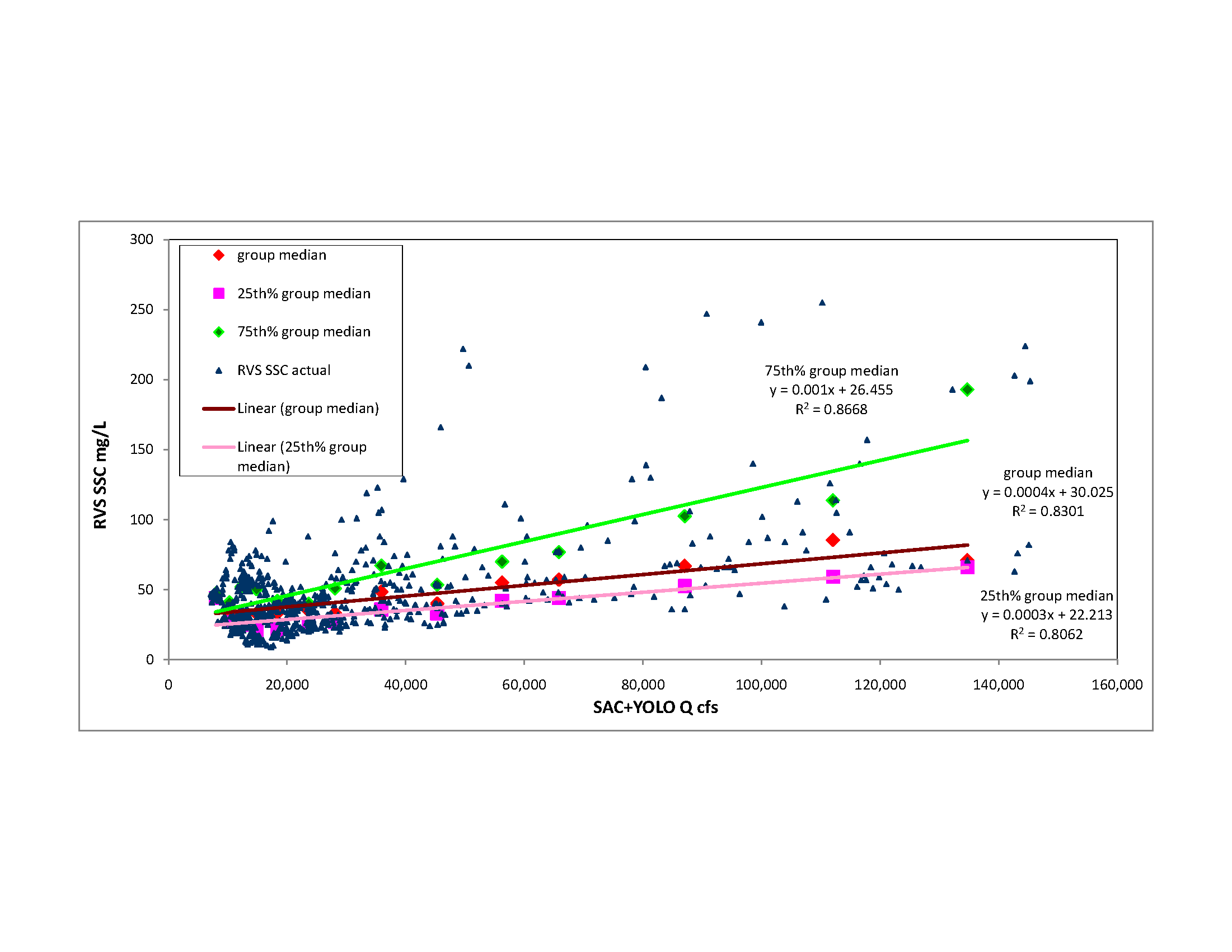

Supplement: Figure S1 — Sediment rating curve for the Sacramento River at Rio Vista, 1998–2002. (TIF) [file pone.0024465.s001.tif]

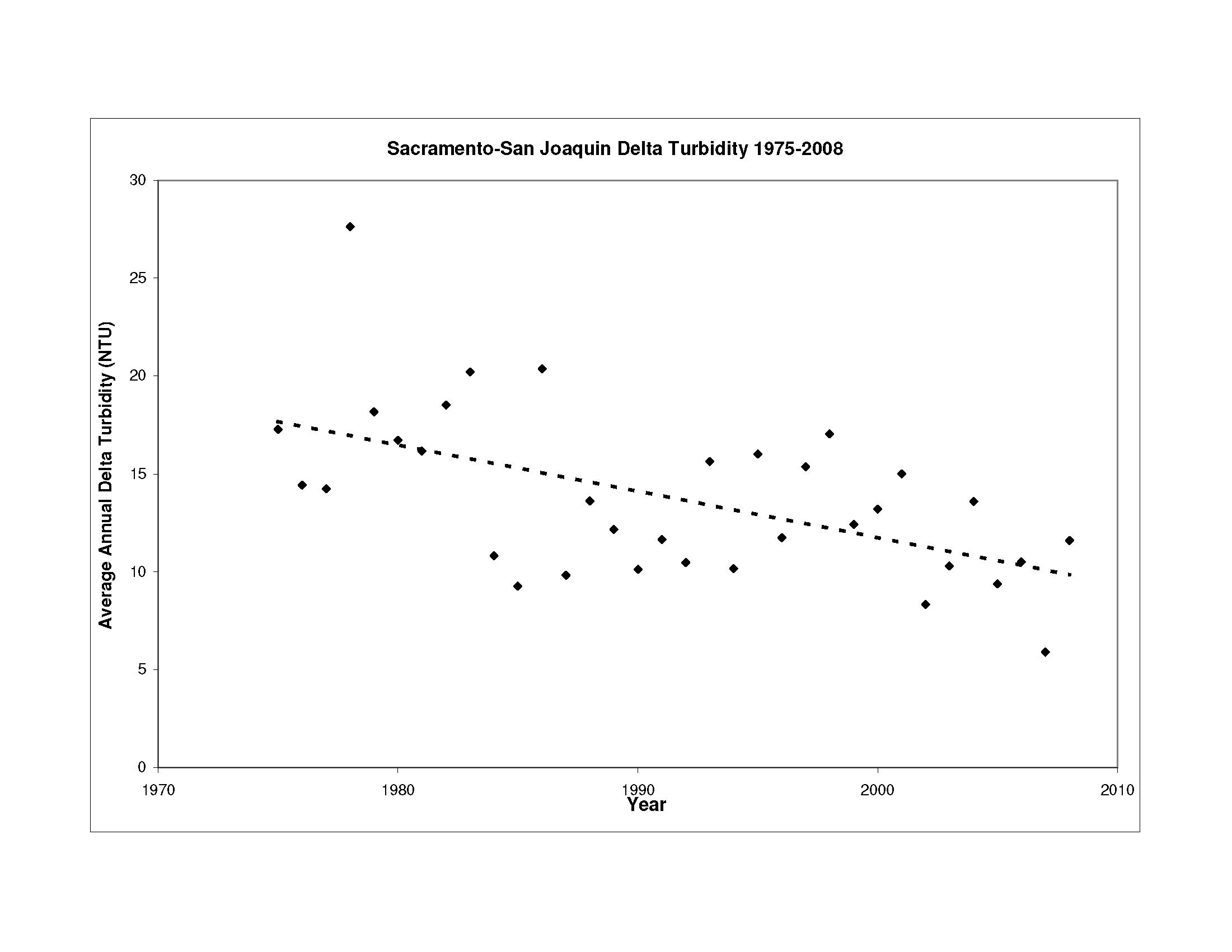

Supplement: Figure S2 — Mean annual turbidity, declining throughout the Sacramento-San Joaquin Delta from 1975–2008. From monthly data provided by California Department of Water Resources, Environmental Monitoring Program. (TIF) [file pone.0024465.s002.tif]

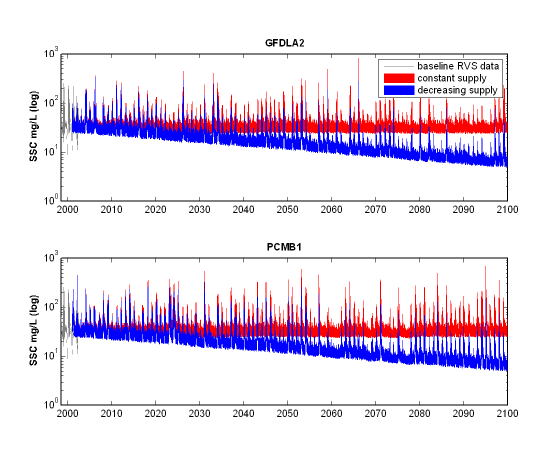

Supplement: Figure S3 — GFDL and PCM scenarios for suspended sediment concentration (SSC) in the Sacramento River at Rio Vista for constant and decreasing sediment supply. Each band represents the interquartile range of SSC. (TIF) [file pone.0024465.s003.tif]
