# Supplementary material for: Projected Evolution of California's San Francisco Bay-Delta-River System in a Century of Climate Change
Source: PLoS One. 2011 Sep 21;6(9):e24465. doi: 10.1371/journal.pone.0024465 (PMC3177826; doi:10.1371/journal.pone.0024465)
Supplement: Methods S1 — Expanded description of methods with supporting references. (RTF) [file pone.0024465.s005.rtf]

Supporting Information
J.E. Cloern et al., “Projected Evolution of California's San Francisco Bay-Delta-River System in a Century of Climate Change”

Expanded Methods
We chose to evaluate two very different scenarios from the GCM projections used in the IPCC Fourth Assessment Report. The PCM-B1 climate scenario portrays the B1 emissions scenario (representing a future where GHG emissions are curtailed by mid-century) as modeled by the Parallel Climate Model, a model with relatively low sensitivity to GHG emissions. The GFDL-A2 climate scenario represents the A2 emissions scenario (corresponding to a future of continually increasing atmospheric greenhouse gases) as modeled by the (medium-sensitivity) NOAA Geophysical Fluid Dynamics Laboratory (GFDL) CM2.1 model. We selected these model-specific scenarios to compare projections of a moderately warmer future with little change in precipitation (PCM1-B1) to a much warmer and drier future (GFDL-A2). While no wetter-on-average scenario was evaluated, precipitation in the PCM-B1 scenario spans nearly the entire range of interannual precipitation variability in the 84-member ensemble (see Fig. 10, [1]). The central tendency of GCM projections is for little precipitation change for smaller warming trends and drier conditions for projections that show a greater warming. In fact, few climate scenarios produced by the AR4 suite of GCMs portray a trend towards more average annual precipitation in California.
While there are multiple sources of uncertainty in this study, the complexity of the systems being studied and the detailed nature of our investigations make the evaluation of a large ensemble of scenarios that would be necessary for a full uncertainty assessment prohibitively difficult. One of the largest sources of uncertainty is apparent in the range of changes contained in the suite of GCM projections used in AR4. By selecting two very different scenarios from this suite, we are able to evaluate how the uncertainty reflected in the differences among global climate models and emissions scenarios manifests in the San Francisco Bay-Delta-watershed system, as reflected by a diverse collection of environmental indicators.
Our general approach was to use linked models, each representing a different component of the system, to propagate the effects of the climate scenarios described above through the Bay-Delta watershed/estuary system. Ultimately we portrayed these effects with a series of environmental indicators representing all components. These indicators were developed for the current century (2010-2099) and for a historical baseline period, defined as 1970-1999 to capture recent historical behavior (1999 is the end year of the “historical” GCM runs—see below). For all indicators, observation-based and model-based indicators were produced for the historical period to allow for model evaluation as well as to provide a baseline against which to assess the scenario projections.
For those indicators based directly on GCM output (air temperature, precipitation, and sea level), “historical” GCM simulations (driven by historical GHG forcings but otherwise unconstrained by observations) by the PCM and GFDL models were used to produce the “model-based” historical indicators. Due to the nature of these indicators, they will not agree on a year-to-year basis with the corresponding observation-based indicators (Fig. 2), and can only be used for model evaluation with regard to overall model bias and variance during the historical period. They also provide a useful baseline against which to compare the corresponding projections.
For all other indicators, the model-based historical indicators are ultimately based on observed meteorological forcings, although they do reflect any errors introduced by the chain of linked models used to produce them. As such, these indicators allow for direct model evaluation by comparison with the corresponding “observation-based” time series, as well as providing a model-based baseline against which to compare the model-based projections.
A detailed description of the individual component methods follows.

Meteorology
Downscaled simulations of 20th and 21st century variations and trends of daily surface-air temperatures and precipitation were used to evaluate potential impacts of climate change on the Bay-Delta and its watershed. For the present evaluations, climate-change scenarios were derived from climate simulations by two global climate (or general circulation) models (GCMs) under one future global greenhouse-gas emissions scenario each. The GCMs used here were the Geophysical Fluid Dynamics Laboratory's (GFDL) CM2.1 coupled ocean-atmosphere GCM [2] and the National Center for Atmospheric Research's Parallel Climate Model (PCM) coupled ocean-atmosphere GCM [3]. Daily values of the climate variables from simulations by the GFDL climate model under A2 (rapidly accelerating) greenhouse-gas emissions, by the PCM climate model under B1 (eventually leveling) greenhouse-gas emissions, and for historical PCM and GFDL model runs (forced using historical emissions) were obtained from the Program for Climate Diagnosis and Intercomparison at the Lawrence Livermore National Laboratory ([4]; www-pcmdi.llnl.gov). The GCM simulations were made on global grids with about 2 to 3º latitude and longitude resolution (about 250 km at the latitude of the Delta), and thus the original GCM scenarios were too spatially coarse for the purposes of this study. The GCM temperatures and precipitation values were downscaled onto a 1/8º latitude-longitude grid over the conterminous US by a method called Constructed Analogs [5]. This statistical downscaling method is applied to each day's simulated climate condition in turn, and is based on fitting a linear combination of historical weather patterns (aggregated from the historical fields of Maurer et al. [6] onto the GCM grid) that best reproduces the GCM pattern for the day. The coefficients necessary to make this linear fit are then applied to high-resolution versions of the weather on the same historical days. The approach ensures that, day by day, the weather simulated by the GCM is faithfully carried down to the 12-km scale, and tends to yield particularly realistic temperature relations across areas with sharp geographic gradients (e.g., from Delta to Central Valley plains; [7]). When applied to the historical record, as a validation exercise, the method reproduces daily temperature variations quite accurately on the 12-km grid given only historical temperatures as observed on the GCM grids as inputs [5], indicating that the downscaled future-climate patterns are likely to also be realistic. The method was applied to climate simulations spanning the period from 1950-2100 (from which the 1970-2099 period used in this study was extracted), to obtain daily, gridded temperature and precipitation patterns over California, from which watershed-average values were extracted. From these outputs, time series of Delta-averaged temperatures and watershed-averaged precipitation (see Fig. 1) were calculated. The corresponding averages based on historical observations were derived from the gridded meteorological dataset of Maurer et al. [8]. 

Sea Level
To investigate sea level trends and extremes, a model was constructed based on historical data and used to project future water levels at the San Francisco Golden Gate tide gage location (see Fig. 1). The model consists of four components:
(a) Synoptic meteorologically-forced sea level fluctuations due to barometric effects (sea level pressure, SLP) and wind stress fluctuations were modeled using a linear regression scheme.  This weather variability, extracted from the GCM simulations, includes local sea level pressure near the San Francisco tide gage as well as the regional wind stress. The linear statistical model is based upon on regression of observed sea level non-tidal residuals from tide gages vs. local SLP and offshore wind stress from the NCAR/NCEP Reanalysis output, 1950–2004, as described in Cayan et al. [7].
(b) ENSO-related monthly-to-interannual time-scale fluctuations contribute the dominant portion of non-anthropogenic sea level variability at seasonal-interannual time scales.  The ENSO component is also amenable to a simple linear model. Assuming that the same mechanisms will operate in the future as during the historical period, the linear relationship between observed monthly Nino 3.4 SST anomalies and the San Francisco station's sea level is used as the ENSO component of the model. ENSO variability was extracted from the 2000–2100 climate model projections using the difference between NINO 3.4 SST and its linear trend over 2000–2100 as a conservative estimate of the ENSO index. The effects of ENSO are represented by area average sea surface temperature (SST) anomalies in the Nino 3.4 region (120ºW–170ºW, 5ºS–5ºN) extracted from the GCMs and scaled to match the standard deviation of the observed Nino 3.4 series for 1961–1990.    
(c) Astronomical tides predicted, at hourly resolution, over the 21st century using harmonic coefficients based upon tidal constituents derived from the historical San Francisco tide gage data [9]. 
(d) Sea level rise was estimated using the Vermeer and Rahmstorf [10] semi-empirical sea level scheme, using global average air temperature from the GCM simulations. 
With these components, simulated sea level at San Francisco was constructed by superimposing the sea level rise onto predicted tides, weather-related sea level anomalies, and ENSO-related sea level fluctuations. The result of the model runs was a series of hourly sea level from 1950 through 2100 (from which the 1970-2099 period used in this study was extracted) for each of the climate scenarios. Historical observations for 1970-1999 were obtained for the Golden Gate tide gage from NOAA (tidesandcurrents.noaa.gov).  

Hydrology and Management
A combination of models was used to simulate the watershed's hydrologic behavior for each scenario. Downscaled meteorological fields (see “Meteorology” above) were used to drive the VIC watershed model [11,12], configured for the Sacramento River and San Joaquin River watersheds using the same parameters applied in several prior studies of the area ([1,13,14]. This resulted in daily estimates of snowpack and unimpaired reservoir inflows for each scenario. A simulation was also performed for the baseline period, driven using historical meteorology ([8] to produce the “observation-based” historical hydrological indicators. Estimates of unimpaired flow at major reservoirs throughout the watershed were obtained from the California-Nevada River Forecast Center (www.cnrfc.noaa.gov) and California Data Exchange Center (CDEC, cdec.water.ca.gov). Data covering the period 1970-1986 were available, allowing watershed total unimpaired runoff and snowmelt fraction of annual runoff to be calculated for this period, providing the observation-based historical time series for those indicators.
The simulations and analyses described in this section were also carried out using a different hydrological model, the Bay-Delta Watershed Model (BDWM), a distributed soil-moisture accounting model of unimpaired hydrology throughout the watershed [15,16]. The results with BDWM were very similar to those produced with VIC, and the conclusions of the overall study appear to be robust with respect to hydrologic model choice.
These inflows were used to drive a model of freshwater management operations—the California Department of Water Resources' CALSIM II model [17]. CALSIM is a management optimization model in which, given inputs of reservoir inflows, a set of freshwater management decisions is computationally determined on a monthly time step that optimally satisfy operational goals and constraints. The results are estimates of managed freshwater flows at points throughout the watershed. CALSIM has been applied in other climate-change studies [18,19,20,21,22], In this study, a new configuration of CALSIM II was used to produce projections for the coming century (2010-2099), and an existing configuration (configured for runs only up to 1994) was used to produce historical estimates (1970-1994).  
A shortcoming of CALSIM is its treatment of groundwater withdrawals, which are allowed in the model to occur at unsustainable levels if other supplies of freshwater are insufficient to meet demands. This must be considered when interpreting results. In particular, simulated unsustainable withdrawal levels are indicative of an inability to meet freshwater demands through other means. In this situation, net depletion of aquifers to meet demand is one possible outcome; measures to reduce demand are another.
Another shortcoming is that CALSIM produces only monthly averaged results. Resulting streamflows at key locations were disaggregated to daily flows by selecting from historical daily flow records at those locations. For each projected future month, an optimally matching historical month was selected, using a combined RMS-error and correlation-coefficient metric to compare projected and simulated historical daily unimpaired flows at upstream sites above the major reservoirs. The corresponding daily flows from the matching historical months at the downstream locations were then scaled to match the CALSIM-simulated monthly averages at those locations. Such approximations are necessary until an operations model with a daily time step is available. Finally, historical and projected stream temperatures were simulated throughout the watershed using the U.S. Bureau of Reclamation's CALSIM-driven stream-temperature model. This model has also been applied in other climate-change studies (e.g., [21]).
In attempting to represent the behavior of a complex freshwater management network like California's in the future, several difficulties are encountered. Projecting freshwater demands is difficult, and for CALSIM, demand scenarios were only available for California's level of development (LOD) in 2000 and that projected for 2020. Thus, historical CALSIM runs use the 2000 LOD, and projections use the 2020 LOD. This necessarily introduces errors into the results, with a key caveat that projections well past 2020 almost certainly underestimate freshwater demands, barring major changes in California water-use patterns. Another important difficulty lies in the fact that freshwater management infrastructure is not static. Representing the numerous historical infrastructure changes is very difficult, and predicting future changes is impossible. Therefore, the CALSIM runs used in this study assume present-day management infrastructure and goals. The results may be interpreted as potential changes which future adjustments to management infrastructure and goals may be designed to help mitigate.

Estuarine Salinity
To project changes in estuarine salinity due to climate change, two models were used. The Uncles-Peterson estuarine model is a 2-dimensional, advective-diffusive, intertidal box model of the San Francisco Estuary with a time step of one day. This model has been applied in several previous studies of the estuary and has been shown to accurately reproduce salinities at weekly to interannual time scales over a wide range of flow regimes [23,24]. Importantly, the U-P model is very economical computationally, enabling the 90-year runs needed to evaluate estuarine variability under the climate-change scenarios The U-P model was driven using daily estuarine inflows derived from CALSIM outputs described above, producing daily salinities along the estuary's axis for both the historical baseline period and for each future scenario. A simulation was also performed for the baseline period using observed inflows (www.water.ca.gov/dayflow) for use in deriving “observation-based” historical salinity values. 
While the U-P simulations provide a representation of the influence of changing hydrology on estuarine salinities over each scenario, the U-P model does not capture the effects of sea level rise on salinity. For this, a separate model, Delft3D [25], was used to parameterize salinity changes due to sea level rise based on a) freshwater inflow rate and b) amount of sea level rise. The Delft3D model of San Francisco Bay [26] is a 3D process-based model covering an area from Point Reyes at sea to the landward boundary of tidal influence near Sacramento on a curvilinear grid with a spatial resolution ranging from 100 to 1000 m. Land with low elevation is included in the model so that sea level rise can result in flooding of currently dry land.   
The Delft3D model is too computationally demanding to evaluate full 90-year scenarios, but it is sophisticated enough to capture salinity changes associated with sea level rise, and is thus complementary to the U-P model. A set of 2-month runs was performed using Delft3D in which a different combination of freshwater inflow rate and sea-level-rise amount characterized each run. The first month of each run was discarded to allow for model spin-up (salinity changes leveled off within a month). For each run, time-averaged salinity values were calculated for points along the estuary's axis corresponding to the U-P model segments. Changes in salinity due to sea level rise were then calculated by subtracting salinities corresponding to no sea level rise and a given inflow rate from salinities corresponding to other values of sea level rise and the same inflow rate. Calculating these changes separately for each inflow rate allowed us to isolate the changes due to sea level rise while still including the effects of inflow rate on those changes. The pattern in plots of salinity changes at all points versus the corresponding baseline salinities was found to be fairly consistent across different values of sea level rise and inflow rate, varying mainly in magnitude. The magnitude of the change was affected by both amount of sea level rise and freshwater inflow. Therefore, bivariate regressions of salinity change on inflow and sea-level-rise amount were performed for binned values of baseline salinity (a different set of regression coefficients for each bin). The resulting coefficients were then used to calculate sea-level-rise-driven salinity increases at points along the estuary's axis, using daily inflows (see “Hydrology and Management” above) and sea-level-rise amounts (see “Sea Level” above) for both the historical and projected periods as the regressors, and using the U-P-simulated salinities to determine which salinity bin's regression coefficients to use. The changes were added to the corresponding U-P salinities, and the final results represent our estimate of salinity changes throughout the estuary due to the combination of upstream hydrologic changes and sea level rise.
Suspended Sediment
To evaluate possible suspended sediment changes under the climate-change scenarios, a rating curve of suspended sediment concentration (SSC) for the Sacramento River at Rio Vista versus river discharge was developed. This location was chosen because it is colocated with an available future scenario variable, streamflow (see “Hydrology and Management” above), and SSC data are available every 15 minutes from 1998-2002 [27]. USGS 15-minute suspended-sediment time series data for the Sacramento River at Rio Vista station covering years 1998-2002 [27] were converted to daily mean SSC and compared with the sum of daily discharge from the Sacramento River at Freeport (USGS station 11455420) and from the Yolo Bypass near Woodland  (USGS station 11447650). A suspended-sediment rating curve (Fig. S1) was developed using linear regression based on group-averaged data [28]. With this approach, an r2 value of 0.83 was obtained using a corresponding prediction equation, an improvement over simple least-squares and multiregression methods. The independent variable (daily discharge) was regressed against the dependent variable (daily SSC) using 15 groups determined using a log transformation on the ranked discharge data. For each discharge group, median, upper quartile (75th percentile), and lower quartile (25th percentile) values were calculated.  Linear regression was used to determine how these values vary with discharge (Fig. S1).  Results using daily-averaged 15-minute SSC data collected during 1998-2002 were more robust (n=689) than for monthly grab sample SSC data collected from 1975-1995 for the Interagency Ecological Program's Environmental Monitoring Program (IEP EMP; n=364).  
For each scenario, calculated daily discharges were used to calculate the daily median and upper and lower quartiles of SSC using the regression equations given on Fig. S1.  One-half of the SSC values would be within the estimated interquartile range.  In the paper, only the annual averages of daily median SSC were used for simplicity.
The sediment delivery from the Sacramento River watershed to the San Francisco Bay has decreased by about one-half between 1957 and 2001 [29]. As these changes in sediment delivery have occurred, the turbidity and associated SSC within the Sacramento-San Joaquin River Delta during the last four decades have also decreased by approximately 40% (Fig. S2). Because of the historical decreasing trend in sediment supply, two sediment-supply scenarios into the future (Fig. S3) were developed. The constant sediment supply scenario was developed assuming the rating curve in Fig. S1 applies in the future. The decreasing sediment supply scenario was developed assuming that SSC decreases at 1.6% yr-1, the Delta-wide average rate of SSC decrease from 1975-2008 (data from the IEP EMP, available at www.water.ca.gov/bdma; Seasonal Kendall test applied [30,31]). In Fig. S3, upper and lower quartiles are plotted to indicate the range in which one-half of the SSC would occur.   
The rating curve was applied to produce a hindcast of SSC, using observed discharges (www.water.ca.gov/dayflow) and the historical trend in sediment delivery. This is presented in Fig. 2 as the “observation-based” time series of SSC during the baseline period, since relatively little observed SSC data exist for that period. The historical “model-based” indicator was produced by applying the rating curve to the CALSIM-based daily discharge estimates (see “Hydrology and Management” above) and using the historical trend in sediment delivery.

Delta Water Temperature
Water temperature data were obtained from the IEP EMP for the Sacramento River at Rio Vista, where water temperatures were collected from May 1983 through September 2002 (1984-1999 annual averages of these data constitute the observation-based historical indicator).  Historical air temperature and insolation data were acquired from the California Irrigation Management Information System (CIMIS, www.cimis.water.ca.gov) at seven locations in the Delta; air temperature data for six more locations were obtained from the IEP. Air temperature and insolation data were averaged spatially, as within-Delta variation of these quantities was small. Daily averages were calculated for all data. To produce water-temperature hindcasts, a longer record of daily air temperatures was required. Data for 1970-2000 were acquired from the California Climate Data Archive (www.calclim.dri.edu/data.html) for five locations around the Delta.  As with the CIMIS data, these data were spatially averaged over the Delta.
Using the historical data, a simple regression was applied to relate the daily-averaged water temperature (T) to the air temperature (Ta) and insolation (R) from the same day and water temperature from the preceding day [32]:

	T(n)=aTa(n)+bT(n-1)+cR(n)+d					(1)

where n is the day on which the temperature is being calculated, and a, b, c and d are coefficients that are determined during the calibration period. The insolation was defined as the average insolation for each Julian day of the year.  
To verify the model, regression coefficients were calculated for equation (1) using the first half of the available historical dataset (the calibration period), then used to force the model during the entire period of coverage for each site (both calibration and verification periods).  The daily average model performed well when compared to measured water temperatures; the r2 value was 0.964 for the verification period.
To project water temperatures for the coming century, the model was calibrated with the entire historical dataset and applied the resulting regression equation to the downscaled climate data, using the mean annual insolation cycle.  Similarly, to hindcast water temperatures for 1970-1999, the model was calibrated with the entire historical dataset and forced with the long-term historical air temperatures and the mean annual insolation cycle, providing the “model-based” historical indicator for Delta water temperature.
Annual average projected temperatures were calculated from the daily projections. These were calculated as a direct average over each calendar year. Although atmospheric forcing alone was enough to produce high model-data correlations on the daily timescale, in the annual average, the modeled and observed values diverge during the late 1990s, as is evident in Fig. 2. These discrepancies occur during years when the flows were high, and comparisons of the error in the projections (annual average of model projections minus the annual average of the observed temperatures) with river flow (Fig. S4) shows that flow explains the discrepancies. More detailed examination of the intra-annual variation during these high flow years (not shown) indicates that in spring/summer seasons with high flows, the warming of the Delta is delayed and, as a result, the summer peak temperature period is shortened, leading to a reduced annual average relative to a prediction based purely on atmospheric forcing. The mechanism driving this flow effect requires further examination, but is most likely due to high flows pushing the high-temperature Delta waters further down-estuary (see [33]). 
The fact that the flow effects develop primarily at high flows mitigates the impact of this error on our climate-based projections, since the projections for unimpaired runoff are either flat (scenario B1) or decline (scenario A2), so the cooling effects of high flows would not be evident over the projections.

Biological Indicators
Delta smelt (Hypomesus transpacificus) is endemic to the San Francisco Estuary [34,35] and is listed as endangered by the state of California and a change in status from threatened to endangered has been deemed warranted under the federal endangered species act.  Thus, maintaining the population of delta smelt has become a key goal in managing the estuary [36].  To assess the effects of climate change on delta smelt, the frequency of mean daily water temperatures above 25 ⁰C based on modeled water temperatures at Rio Vista (see “Delta Water Temperature” above) determined for Rio Vista.  Rio Vista is within one tidal excursion of a large portion of the remaining delta smelt habitat in the tidal portion of the Sacramento River. Bennett [35] indicated that temperatures above 25 ⁰C were likely lethal to delta smelt.  Swanson et al. [37] obtained a laboratory-derived acute lethal limit of 25 ⁰C (for fish acclimated to 17 ⁰C, [37]).  Nobriga et al. [38] found that catch of delta smelt began decreasing at temperatures above 20 ° C and became almost zero at 25 °C suggesting avoidance of stressful conditions or high mortality.  Finally, recent observations of control groups of delta smelt held as part of mark-recapture studies (Castillo et al., 2010) indicated that exposure of well fed, unstressed hatchery-reared delta smelt to ambient water temperatures that exceeded 25°C for several days resulted in a rapid decline in survival.  This mortality was not reversed when ambient water temperature declined below 25°C, suggesting major and irreversible physiological impairment.  Thus, a mean daily temperature of 25°C, with temperatures above 25°C for much of the day, seems a reasonable threshold for expecting high mortality of fish in the wild.
Winter-run Chinook salmon (Oncorhynchus tshawytscha) is endemic to the Sacramento River system of California and is listed as endangered under both state and federal endangered species legislation [34].  Most of the population is restricted to the portion of the Sacramento River downstream of Keswick Reservoir, which is a regulating reservoir for Shasta Reservoir.  These reservoirs prevent access to most of the historical habitat of the species.  Providing appropriate temperatures to maintain the species while maintaining water deliveries for human purposes is a major goal of water management of Shasta Reservoir.  Winter-run Chinook salmon begin spawning in the spring.  Developing embryos and pre-emergent fry are expected to be in the gravel from May through October.  The effects of climate change on winter-run Chinook salmon were assessed by comparing projected mean monthly water temperatures from a model developed by the U.S. Bureau of Reclamation (see “Hydrology and Management” above) for the period May-October against a threshold of 16°C, which would result in high mortality of eggs and pre-emergent fry.  This is likely a conservative comparison since in a month with a mean of 16°C approximately half the days would have higher temperatures.  Comparisons were made for the Sacramento River at Balls Ferry, which is at the lower end of the spawning reach. Historical temperature data were obtained for 1991-1999 from CDEC, processed to remove unreasonable values, and used to produce the corresponding observation-based historical indicator. Stream temperature data from the historical run of the stream temperature model (1970-1994—see “Hydrology and Management” above) were used to produce the model-based historical indicator.
Sacramento splittail (Pogonichthys macrolepidotus) is a large cyprinid, endemic to the San Francisco estuary and watershed [34,39].  Splittail are true floodplain spawners and production of strong year classes of young splittail is associated with flooding of Sutter and Yolo bypasses.  The bypasses are floodways designed to protect Sacramento and other urban areas from flooding.  Yolo bypass has been extensively studied and is now known to provide benefits to native fishes, including Chinook salmon and splittail [40,41].  Floodplains must stay continuously flooded for a minimum of about 30 days [42] for splittail to successfully spawn.  Longer periods of inundation result in greater production of young splittail [39].  Yolo Bypass provides appropriate spawning conditions at flows above about 4,000 cfs.  The frequency of maximum continuous floods with durations of 30 days (flows continuously above 4,000 cfs) or more was counted.

Analysis
The trend slope for each time series (portrayed in Fig. 2) was calculated using the approach of Theil [43] and Sen [44]. This Theil-Sen slope is simply the median slope of the lines joining all pairs of data points in the series. Note that Theil-Sen slopes sometimes differ substantially from linear trend estimates, just as the median and mean of a number set sometimes differ substantially. The Mann-Kendall test [30,45] is often used as a nonparametric test for the statistical significance of these slopes. But hydrological and other time series frequently display serial correlation. Serial correlation increases the probability of detecting a significant trend and leads to a disproportionate rejection of the null hypothesis of no trend. Trend significance was therefore determined using the modified Mann-Kendall approach of Yue and Pilon [46], which corrects for serial correlation more effectively than older pre-whitening methods. The confidence interval on the trend is calculated using the method described by Sen [44]. Autocorrelation was indeed quite large for some annual series, up to 0.65 for the sea level A2 scenario. Calculations were carried out using the zyp package [47] for R [48].


References

1. Cayan DR, Maurer EP, Dettinger MD, Tyree M, Hayhoe K (2008) Climate change scenarios for the California region. Climatic Change 87: 21-42.
2. Delworth TL, Broccoli AJ, Rosati A, Stouffer RJ, Balaji V, et al. (2006) GFDL's CM2 Global Coupled Climate Models. Part I: Formulation and Simulation Characteristics. Journal of Climate 19: 643-674.
3. Washington WM, Weatherly JW, Meehl GA, Semtner Jr AJ, Bettge TW, et al. (2000) Parallel climate model (PCM) control and transient simulations. Climate Dynamics 16: 755-774.
4. Meehl GA, Covey C, Delworth T, Latif M, McAvaney B, et al. (2007) THE WCRP CMIP3 Multimodel Dataset: A New Era in Climate Change Research. Bulletin of the American Meteorological Society 88: 1383.
5. Hidalgo HG, Dettinger MD, Cayan DR, California Energy Commission. Public Interest Energy Research., Scripps Institution of Oceanography., et al. (2008) Downscaling with constructed analogues : daily precipitation and temperature fields over the United States : PIER final project report. Sacramento, Calif.: California Energy Commission. x, 48 p. p.
6. Maurer EP, Wood AW, Adam JC, Lettenmaier DP, Nijssen B (2002) A long-term hydrologically based dataset of land surface fluxes and states for the conterminous United States. Journal of Climate 15: 3237-3251.
7. Cayan D, Tyree M, Dettinger M, Hidalgo H, Das T, et al. (2009) Climate change scenarios and sea level rise estimates for California 2008 Climate Change Scenarios Assessment: California Energy Comission Report CEC-500-2009-014-D. 62 p.
8. Maurer EP, Wood AW, Adam JC, Lettenmaier DP, Nijssen B (2002) A long-term hydrologically-based data set of land surface fluxes and states for the conterminous United States. J Clim 15: 3237–3251.
9. Zetler BD, Flick RE (1985) Predicted Extreme High Tides for Mixed-Tide Regimes. Journal of Physical Oceanography 15: 357-359.
10. Vermeer M, Rahmstorf S (2009) From the Cover: Global sea level linked to global temperature. Proceedings of the National Academy of Sciences 106: 21527-21532.
11. Liang X, Lettenmaier DP, Wood EF, Burges SJ (1994) A simple hydrologically based model of land surface water and energy fluxes for general circulation models. J Geophys Res 99: 14415–14428.
12. Cherkauer KA, Bowling LC, Lettenmaier DP (2003) Variable infiltration capacity cold land process model updates. Global Plan Change 38: 151–159.
13. Barnett TP, Pierce DW, Hidalgo HG, Bonfils C, Santer BD, et al. (2008) Human-induced changes in the hydrology of the western United States. Science 319: 1080-1083.
14. Maurer EP, Hidalgo HG, Das T, Dettinger MD, Cayan DR (2010) The utility of daily large-scale climate data in the assessment of climate change impacts on daily streamflow in California. Hydrol Earth Syst Sci 14: 1125-1138.
15. Knowles N (2000) Modeling the Hydroclimate of the San Francisco Bay-Delta Estuary and Watershed [Ph. D]. La Jolla, CA: Scripps Institution of Oceanography, University of California - San Diego.
16. Knowles N, Cayan D (2004) Elevational dependence of projected hydrologic changes in the San Francisco Estuary and Watershed. Climatic Change 62: 319-336.
17. Draper AJ, Munevar A, Arora SK, Reyes E, Parker NL, et al. (2004) CalSim: Generalized Model for Reservoir System Analysis. Journal of Water Resources Planning and Management 130: 480-489. California Energy Commission, PIER Energy-Related Environmental Research. CEC-500-2005-053.
18. Brekke LD, Miller NL, Bashford KE, Quinn NWT, Dracup JA (2004) Climate change impacts uncertainty for water resources in the San Joaquin River Basin, California. Journal of the American Water Resources Association 40: 149-164.
19. Dracup JA, S. Vicuna, R. Leonardson, L. Dale, M. Hanneman. (2005) Climate Change and Water Supply Reliability.
20. Vicuna S, Maurer EP, Joyce B, Dracup JA, Purkey D (2007) The Sensitivity of California Water Resources to Climate Change Scenarios. Journal of the American Water Resources Association 43(2): 482-498.
21. Anderson J, Chung FI, Anderson M, Brekke L, Easton D, et al. (2008) Progress on incorporating climate change into management of California's water resources. Climatic Change 87, suppl. 1: 91– 108.
22. Brekke LD, Maurer EP, Anderson JD, Dettinger MD, Townsley ES, et al. (2009) Assessing reservoir operations risk under climate change. Water Resources Research 45: W04411.
23. Peterson D, Cayan D, Dileo J, Noble M, Dettinger M (1995) The role of climate in estuarine variability. American Scientist 83: 58-67.
24. Knowles N, Cayan D, Peterson DH, Uncles RJ (1998) Simulated Effects of Delta Ouflow on the Bay: 1998 Compared to Other Years. Interagency Ecological Program Newsletter: 29-31.
25. Lesser GR, Roelvink JA, van Kester JATM, Stelling GS (2004) Development and validation of a three-dimensional morphological model. Coastal Engineering 51: 883-915.
26. Elias E, Van der Wegen M, Roelvink JA Calibration and validation of a 3D process-based model for San Francisco Bay and Delta. in preparation.
27. Wright SA, Schoellhamer DH (2005) Estimating sediment budgets at the interface between rivers and estuaries with application to the Sacramento-San Joaquin River Delta. Water Resources Research 41: W09428.
28. Glysson DG (1987) Sediment-transport curves. U.S. Geological Survey Open File Report, 87-218.
29. Wright SA, Schoellhamer DH (2004) Trends in the Sediment Yield of the Sacramento River, California, 1957 - 2001. San Francisco Estuary and Watershed Science 2.
30. Kendall MG (1975) Rank Correlation Methods. Oxford Univ. Press, New York.
31. Helsel DR, Hirsch RM (1992) Statistical methods in water resources. Elsevier, Amsterdam.
32. Wagner R, Stacey MT, Brown L, Dettinger M (2011) Statistical models of temperature in the Sacramento-San Joaquin Delta under climate-change scenarios and ecological implications. Estuaries and Coasts 34: 544-556.
33. Monismith SG, Hench JL, Fong DA, Nidzieko NJ, W.E. F, et al. (2009) Thermal variability in a tidal river. Estuaries and Coasts 32(1): 100-110.
34. Moyle PB (2002) Inland fishes of California. Berkeley: University of California Press. xv, 502 p. p.
35. Bennett WA (2005) Critical assessment of the delta smelt population in the San Francisco Estuary, California. San Francisco Estuary and Watershed Science 3.
36. Sommer T, Armor C, Baxter R, Breuer R, Brown L, et al. (2007) The Collapse of Pelagic Fishes in the Upper San Francisco Estuary. Fisheries 32: 270-277.
37. Swanson C, Reid T, Young PS, Cech Jr JJ (2000) Comparative environmental tolerances of threatened delta smelt (Hypomesus transpacificus) and introduced wakasagi (H. nipponensis) in an altered California estuary. Oecologia 123: 384-390.
38. Nobriga ML, Sommer TR, Feyrer F, Fleming K (2008) Long-Term Trends in Summertime Habitat Suitability for Delta Smelt (Hypomesus transpacificus). San Francisco Estuary and Watershed Science 6.
39. Moyle PB, Baxter RD, Sommer T, Foin TC, Matern SA (2004) Biology and population dynamics of Sacramento splittail (Pogonichthys macrolepidotus) in the San Francisco Estuary: a review. San Francisco Estuary and Watershed Science 2: Article 3.
40. Feyrer F, Sommer T, Harrell W (2006) Managing floodplain inundation for native fish: production dynamics of age-0 splittail (Pogonichthys macrolepidotus) in California's Yolo Bypass. Hydrobiologia 573: 213-226.
41. Sommer T, Harrell B, Nobriga M, Brown R, Moyle P, et al. (2001) California's Yolo Bypass: Evidence that flood control can be compatible with fisheries, wetlands, wildlife, and agriculture. Fisheries 26: 6-16.
42. Sommer T, Baxter R, Herbold B (1997) Resilience of Splittail in the Sacramento–San Joaquin Estuary. Transactions of the American Fisheries Society 126: 961-976.
43. Theil H (1950) A rank-invariant method of linear and polynomial regression analysis. I Nederlands Akad Wetensch Proc 53: 386-392.
44. Sen PK (1968) Estimates of the Regression Coefficient Based on Kendall's Tau. Journal of the American Statistical Association 63: 1379-1389.
45. Mann H (1945) Nonparametric tests against trend. Econometrica: Journal of the Econometric Society 13: 245-259.
46. Yue S, Pilon P, Phinney B, Cavadias G (2002) The influence of autocorrelation on the ability to detect trend in hydrological series. Hydrological Processes 16: 1807-1829.
47. Bronaugh D, Werner A (2009) [for the Pacific Climate Impacts Consortium] zyp: Zhang + Yue-Pilon trends package. R package version 09-1.
48. R Development Core Team (2010) R: A language and environment for statistical computing. Vienna, Austria.
